# Risk Factors Associated With SARS-CoV-2 Infection Among Farmworkers in Monterey County, California

**DOI:** 10.1001/jamanetworkopen.2021.24116

**Published:** 2021-09-15

**Authors:** Ana M. Mora, Joseph A. Lewnard, Katherine Kogut, Stephen A. Rauch, Samantha Hernandez, Marcus P. Wong, Karen Huen, Cynthia Chang, Nicholas P. Jewell, Nina Holland, Eva Harris, Maximiliano Cuevas, Brenda Eskenazi

**Affiliations:** 1 Center for Environmental Research and Children’s Health, School of Public Health, University of California, Berkeley; 2 Central American Institute for Studies on Toxic Substances, Universidad Nacional, Heredia, Costa Rica; 3 Center for Computational Biology, College of Engineering, University of California, Berkeley; 4 Division of Epidemiology, School of Public Health, University of California, Berkeley; 5 Division of Infectious Diseases & Vaccinology, School of Public Health, University of California, Berkeley; 6 Department of Medical Statistics, London School of Hygiene and Tropical Medicine, London, United Kingdom; 7 Division of Biostatistics, School of Public Health, University of California, Berkeley; 8 Clinica de Salud del Valle de Salinas, Salinas, California

## Abstract

**Importance:**

Essential workers in agriculture and food production have been severely affected by the ongoing COVID-19 pandemic.

**Objective:**

To identify risk factors associated with SARS-CoV-2 infection among farmworkers in California.

**Design, Setting, and Participants:**

This cross-sectional study invited farmworkers in California’s Salinas Valley (Monterey County) receiving transcription-mediated amplification (TMA) tests for SARS-CoV-2 infection at federally qualified community clinics and community sites to participate. Individuals were eligible if they were not pregnant, were 18 years or older, had conducted farmwork since the pandemic started, and were proficient in English or Spanish. Survey data were collected and SARS-CoV-2 tests were conducted among participants from July 16 to November 30, 2020.

**Exposures:**

Sociodemographic, household, community, and workplace characteristics.

**Main Outcomes and Measures:**

TMA- and immunoglobulin G (IgG)–positive SARS-CoV-2 infection.

**Results:**

A total of 1107 farmworkers (581 [52.5%] women; mean [SD] age, 39.7 [12.6] years) were included in these analyses. Most participants were born in Mexico (922 [83.3%]), were married or living with a partner (697 [63.0%]), and worked in the fields (825 [74.5%]). Overall, 118 of 911 (13.0%) had a positive result on their TMA test for SARS-CoV-2 infection, whereas 201 of 1058 (19.0%) had antibody evidence of infection. In multivariable analyses accounting for recruitment venue and enrollment period, the incidence of TMA-positive SARS-CoV-2 infection was higher among those with lower than primary school–level education (adjusted relative risk [aRR], 1.32; 95% CI, 0.99-1.76; non–statistically significant finding), who spoke an Indigenous language at home (aRR, 1.30; 95% CI, 0.97-1.73; non–statistically significant finding), who worked in the fields (aRR, 1.60; 95% CI, 1.03-2.50), and who were exposed to a known or suspected COVID-19 case at home (aRR, 2.98; 95% CI, 2.06-4.32) or in the workplace (aRR, 1.59; 95% CI, 1.18-2.14). Positive results on IgG tests for SARS-CoV-2 infection were more common among those who lived in crowded housing (aRR, 1.23; 95% CI, 0.98-1.53; non–statistically significant finding), with children aged 5 years or younger (aRR, 1.40; 95% CI, 1.11-1.76), with unrelated roommates (aRR, 1.40; 95% CI, 1.19-1.64), and with an individual with known or suspected COVID-19 (aRR, 1.59; 95% CI, 1.13-2.24). The risk of IgG positivity was also higher among those with body mass index of 30 or greater (aRR, 1.65; 95% CI, 1.01-2.70) or diabetes (aRR, 1.31; 95% CI, 0.98-1.75; non–statistically significant finding).

**Conclusions and Relevance:**

In this cross-sectional study of farmworkers in California, both residential and workplace exposures were associated with SARS-CoV-2 infection. Urgent distribution of COVID-19 vaccines and intervention on modifiable risk factors are warranted given this population’s increased risk of infection and the essential nature of their work.

## Key Points

**Question:**

What are the risk factors associated with SARS-CoV-2 infection among farmworkers in California?

**Findings:**

In this cross-sectional study of 1107 farmworkers, both household and workplace risk factors, including living with children aged 5 years or younger or unrelated roommates and living or working with an individual with known or suspected COVID-19, were associated with positive results on transcription-mediated amplification tests and immunoglobulin G tests for SARS-CoV-2 infection.

**Meaning:**

These findings suggest that urgent distribution of vaccines to farmworkers and intervention on modifiable risk factors for SARS-CoV-2 infection are warranted given this population’s increased risk and the essential nature of their work.

## Teaser

This cross-sectional study identifies risk factors associated with SARS-CoV-2 infection among farmworkers in California.

## Introduction

Essential workers in agriculture and food production have been severely affected by the ongoing COVID-19 pandemic.^1^ In Monterey County, California, we observed a 4-fold higher SARS-CoV-2 test positive fraction among farmworkers tested in community clinics between June and November 2020 than in the county population at large (22% vs 6%).^2,3^ In addition, recent studies have shown that agricultural and food workers in California experienced a 39% higher risk of all-cause death from March to October 2020 than during the same period in 2019, a greater increase than any other occupational group^4^; for workers with Latino backgrounds, the increase in all-cause mortality was 60%.^5^

Widely reported COVID-19 outbreaks among workers involved in food processing facilities have drawn attention to circumstances potentially placing agricultural and food workers at risk for SARS-CoV-2 infection, including poor hygienic conditions, medical leave policies, and residential crowding.^6,7^ However, specific exposures accounting for the high risk of SARS-CoV-2 infection among farmworkers remain poorly understood, and there is uncertainty about what strategies can be undertaken to reduce risk of infection in this population.^8^

Agricultural work is one of the lowest-paid occupations of the US economy, with 29% of full-time workers earning an annual income of less than $26 200 for a family of 4.^9^ Most US farmworkers are Latino (83%),^10^ and approximately one-third live in crowded housing,^10,11,12^ much of which is of substandard quality.^12,13^ In California, at least half of farmworkers are believed to be undocumented,^10^ which could further lead to labor exploitation and fewer workplace protections. In this study, we assessed sociodemographic, household, community, and workplace factors associated with SARS-CoV-2 infection in a population of more than 1000 farmworkers working in Monterey County, California.

## Methods

The protocols for this cross-sectional study were approved by the Office for the Protection of Human Subjects at University of California, Berkeley. All participants provided written informed consent. This study followed the Strengthening the Reporting of Observational Studies in Epidemiology (STROBE) reporting guideline.

### Study Setting

The Salinas Valley, located within Monterey County, California, is home to an agricultural workforce of approximately 50 000 resident farmworkers, with an additional 40 000 seasonal workers supporting the peak summer and fall seasons.^12^ Clinica de Salud del Valle de Salinas (CSVS), a federally qualified community health center, is the main health care system for Monterey County’s farmworkers and their families, with a network of 12 clinics throughout the valley that serve a low-income, primarily Spanish-speaking population of approximately 50 000 individuals.

### SARS-CoV-2 Testing

Testing for SARS-CoV-2 infection at CSVS began June 15, 2020, and was offered to all individuals regardless of exposure, symptoms, documentation, or health insurance status. Medical personnel collected oropharyngeal specimens for detection of SARS-CoV-2 RNA via the qualitative Hologic/Aptima nucleic acid transcription-mediated amplification (TMA) assay. TMA comprises the isothermal amplification of SARS-CoV-2 ribosomal RNA by reverse transcriptase and subsequent generation of numerous transcripts by RNA polymerase.^14^ Testing was conducted on clinic premises or at community sites, including low-income housing, agricultural fields, and CSVS-run community health fairs.

### Study Enrollment

Between July 16 and November 30, 2020, we invited farmworkers (whom we considered to include anyone employed in the agricultural sector) receiving care or getting tested for SARS-CoV-2 infection at CSVS clinics and community sites to participate in our study. We posted flyers about the study at the clinics and around town and provided study information to community groups and growers. Farmworkers were eligible for participation if they were not pregnant, were aged 18 years or older, had conducted farmwork within the 2 weeks preceding their testing date, and were sufficiently proficient in English or Spanish to give consent and complete study procedures. Beginning October 5, we enrolled any individual who had engaged in agricultural work at any time since March 2020 because the growing season was ending.

We enrolled a total of 1115 farmworkers. We excluded from analyses 8 farmworkers who did not provide blood samples or were not employed as farmworkers at the time of enrollment, leaving a total of 1107 participants.

### Study Procedures

After the participant completed the SARS-CoV-2 TMA test and consented to participate in the study, the study team obtained a blood sample by venipuncture for testing of anti–SARS-CoV-2 antibody status. We then measured height and weight using a digital scale. The study team administered a 45-minute computer-guided questionnaire by telephone in Spanish or English within 2 days before (for preconsented participants) or after the enrollment visit but before SARS-CoV-2 testing results were available to avoid recall bias. The questionnaire gathered information on sociodemographic characteristics, risk factors for SARS-CoV-2 infection, and consequences of the pandemic on daily life and well-being. Participants received $50 on completion of all data collection activities.

Blood specimens were stored immediately at 4 to 7 °C and centrifuged and aliquoted within 48 hours following collection. We used an in-house enzyme-linked immunosorbent assay (ELISA) to measure immunoglobulin G (IgG) reactivity to the SARS-CoV-2 spike and receptor binding domain proteins, as described previously.^2^

### Statistical Analysis

We examined risk factors associated with TMA-positive and IgG-positive results for SARS-CoV-2 infection separately. Analyses examining risk factors for positive results on TMA tests included participants who worked in agriculture in the 2 weeks preceding enrollment (n = 911); analyses for positive results on IgG tests included all farmworkers who provided a blood sample (n = 1058).

We performed bivariate analyses for a wide range of sociodemographic, household, community, and work-related characteristics known or suspected to be associated with SARS-CoV-2 infection (Table 1, Table 2, Table 3, and Table 4; eTables 1-4 in Supplement 1) and assessed correlations between these characteristics (eFigure in Supplement 1). We included covariates in multivariable models if there were more than 5 TMA positive or IgG positive cases in each category, respectively, and a χ^2^ or *t* test with *P* < .20 in bivariate analyses. Categorical risk factors were modeled as shown in Table 1, Table 2, Table 3, and Table 4, except for language spoken at home (modeled as Indigenous language spoken at home, yes or no) and working in the fields (yes or no). Age, years in the US, and household size were modeled as continuous variables. We did not consider specific agricultural crops in multivariable analyses because farmworkers reported working in a variety of them. We used backward stepwise elimination (with a threshold of *P* < .10) to select covariates for inclusion in final models.

**Table 1.** Sociodemographic and Health-Related Risk Factors for TMA and IgG Positivity Among Farmworkers, Monterey County, 2020

Attribute	Individuals, No. (%)^a^				
	All enrolled (N = 1107)	SARS-CoV-2 infection			
		TMA positive (n = 911)		IgG positive (n = 1058)	
		Yes (n = 118)	No (n = 793)	Yes (n = 201)	No (n = 857)
Recruitment site					
Clinics	561 (50.7)	95 (18.4)^b^	420 (81.6)	97 (18.4)	429 (81.6)
Community outreach	546 (49.3)	23 (5.8)	373 (94.2)	104 (19.5)	428 (80.5)
Agricultural work in the preceding 2 weeks					
No	193 (17.4)	NA	NA	45 (23.3)^c^	148 (76.7)
Yes	914 (82.6)	118 (13.0)	793 (87.0)	156 (18.0)	709 (82.0)
Sex					
Female	581 (52.5)	60 (13.0)	400 (87.0)	99 (18.1)	448 (81.9)
Male	526 (47.5)	58 (12.9)	393 (87.1)	102 (20.0)	409 (80.0)
Age, y					
Mean (SD)	39.7 (12.6)	39.6 (11.0)	39.6 (12.4)	39.6 (12.3)	39.6 (12.6)
18-29	275 (24.8)	27 (12.0)	198 (88.0)	43 (16.3)^c^	220 (83.7)
30-39	271 (24.5)	29 (12.9)	195 (87.1)	59 (22.5)	203 (77.5)
40-49	297 (26.8)	42 (16.4)	214 (83.6)	59 (20.8)	225 (79.2)
50-59	198 (17.9)	16 (10.2)	141 (89.8)	27 (14.5)	159 (85.5)
≥60	66 (6.0)	4 (8.2)	45 (91.8)	13 (20.6)	50 (79.4)
Education					
≤Primary school	488 (44.1)	63 (15.4)^b^	346 (84.6)	100 (21.2)^d^	372 (78.8)
>More than primary school	618 (55.8)	55 (11.0)	446 (89.0)	101 (17.3)	484 (82.7)
No answer	1 (0.1)	0	1 (100.0)	0	1 (100.0)
Marital status					
Not married or living as married	409 (36.9)	50 (15.3)^d^	277 (84.7)	69 (17.8)	319 (82.2)
Married or living as married	697 (63.0)	67 (11.5)	516 (88.5)	132 (19.7)	537 (80.3)
No answer	1 (0.1)	1 (100.0)	0	0	1 (100.0)
Annual household income, $					
<25 000	557 (50.3)	66 (14.5)	390 (85.5)	101 (18.8)	435 (81.2)
≥25 000	494 (44.6)	48 (11.6)	367 (88.4)	86 (18.5)	380 (81.5)
No answer	56 (5.1)	4 (10.0)	36 (90.0)	14 (25.0)	42 (75.0)
Language spoken at home					
English	57 (5.1)	0 (0.0)	42 (100.0)	12 (21.8)	43 (78.2)
Indigenous	110 (9.9)	22 (22.7)	75 (77.3)	23 (21.5)	84 (78.5)
Spanish	940 (84.9)	96 (12.4)^b^	676 (87.6)	166 (18.5)	730 (81.5)
No answer	0	0	0	0	0
Country of birth					
Mexico	922 (83.3)	104 (13.5)^d^	669 (86.5)	163 (18.4)	721 (81.6)
United States	141 (12.7)	7 (7.0)	93 (93.0)	31 (23.0)	104 (77.0)
Other	44 (4.0)	7 (18.4)	31 (81.6)	7 (18.0)	32 (82.1)
Time in United States, y					
Mean (SD)	21.3 (11.1)	20.2 (10.9)	21.0 (11.2)	21.3 (10.6)	21.2 (11.3)
<15	262 (23.7)	38 (16.5)^d^	193 (83.5)	46 (18.0)^d^	209 (82.0)
15-19	191 (17.3)	17 (10.4)	147 (89.6)	44 (24.0)	139 (76.0)
20-29	296 (26.7)	34 (13.5)	218 (86.5)	49 (17.4)	232 (82.6)
≥30	216 (19.5)	22 (13.4)	142 (86.6)	31 (15.3)	172 (84.7)
Entire life	141 (12.7)	7 (7.0)	93 (93.0)	31 (23.0)	104 (77.0)
No answer	1 (0.1)	0	0	0	1 (100.0)
Community of residence					
Salinas	486 (43.9)	40 (10.4)^b^	343 (89.6)	99 (21.2)^b^	369 (78.8)
Greenfield	315 (28.5)	56 (19.8)	227 (80.2)	63 (21.2)	234 (78.8)
Other town	306 (27.6)	22 (9.0)	223 (91.0)	39 (13.3)	254 (86.7)
Smoking					
Never	899 (81.2)	99 (13.6)	630 (86.4)	157 (18.4)	698 (81.6)
Former	158 (14.3)	16 (11.4)	124 (88.6)	36 (23.4)	118 (76.6)
Current	49 (4.4)	3 (7.3)	38 (92.7)	8 (16.7)	40 (83.3)
No answer	1 (0.1)	0	1 (100.0)	0	1 (100.0)
BMI					
Mean (SD)	29.7 (5.5)	29.2 (4.7)	29.7 (5.6)	30.4 (5.4)^b^	29.4 (5.5
Underweight or normal, <25	197 (17.8)	18 (10.8)	149 (89.2)	24 (12.5)^b^	168 (87.5)
Overweight, 25.0-29.9	421 (38.0)	45 (13.0)	302 (87.0)	75 (18.6)	329 (81.4)
Obesity, ≥30	461 (41.6)	49 (13.1)	326 (86.9)	95 (21.7)	342 (78.3)
Not collected	28 (2.5)	6 (27.3)	16 (72.7)	7 (28.0)	18 (72.0)
Self-reported hypertension					
No	954 (86.2)	106 (13.5)	680 (86.5)	171 (18.7)	744 (81.3)
Yes	149 (13.5)	12 (9.8)	110 (90.2)	29 (20.9)	110 (79.1)
No answer	4 (0.4)	0	3 (100.0)	1 (25.0)	3 (75.0)
Self-reported diabetes					
No	977 (88.3)	109 (13.6)^d^	694 (86.4)	172 (18.4)^d^	762 (81.6)
Yes	126 (11.4)	9 (8.6)	96 (91.4)	28 (23.3)	92 (76.7)
No answer	4 (0.4)	0	3 (100.0)	1 (25.0)	3 (75.0)

Abbreviations: BMI, body mass index (calculated as weight in kilograms divided by height in meters squared); IgG, immunoglobulin G; NA, not applicable; TMA, transcription-mediated amplification.

^a^ Missing entries were excluded from bivariate analyses.

^b^ *P* < .05.

^c^ *P* < .10.

^d^ *P* < .20.

**Table 2.** Household and Community Risk Factors for TMA and IgG Positivity Among Farmworkers, Monterey County, 2020

Attribute	Individuals, No. (%)^a^				
	All enrolled (N = 1107)	SARS-CoV-2 infection			
		TMA positive (n = 911)		IgG positive (n = 1058)	
		Yes (n = 118)	No (n = 793)	Yes (n = 201)	No (n = 857)
Type of housing					
House	522 (47.2)	60 (13.5)	383 (86.5)	101 (20.6)	389 (79.4)
Apartment	481 (43.5)	48 (12.9)	324 (87.1)	85 (18.2)	383 (81.8)
Hotel or motel	37 (3.3)	6 (16.7)	30 (83.3)	5 (13.5)	32 (86.5)
Trailer or mobile home	43 (3.9)	3 (7.7)	36 (92.3)	8 (19.5)	33 (80.5)
Other	24 (2.2)	1 (4.8)	20 (95.2)	2 (9.1)	20 (90.9)
Household size					
Mean (SD)	5.5 (2.5)	5.5 (2.4)	5.4 (2.3)	5.9 (2.6)^b^	5.4 (2.6)
0 others	12 (1.1)	2 (18.2)	9 (81.8)	3 (25.0)^b^	9 (75.0)
1-3 others	397 (35.9)	41 (12.4)	290 (87.6)	58 (15.3)	321 (84.7)
4-6 others	512 (46.3)	51 (12.3)	363 (87.7)	93 (19.1)	393 (80.9)
≥7 others	186 (16.8)	24 (15.5)	131 (84.5)	47 (26.0)	134 (74.0)
Children <18 y living in the home					
No	277 (25.0)	28 (11.9)	207 (88.1)	43 (16.0)^c^	225 (84.0)
Yes	829 (74.9)	90 (13.3)	585 (86.7)	157 (19.9)	632 (80.1)
No answer	1 (0.1)	0	1 (100.0)	1 (100.0)	0
Children ≤5 y living in the home					
No	699 (63.1)	79 (13.7)	498 (86.3)	110 (16.4)^b^	559 (83.6)
Yes	408 (36.9)	39 (11.7)	295 (88.3)	91 (23.4)	298 (76.6)
Children attending school or daycare					
No	1018 (92.0)	105 (12.6)	726 (87.4)	184 (18.9)	792 (81.1)
Yes	85 (7.7)	12 (15.8)	64 (84.2)	16 (20.5)	62 (79.5)
No answer	4 (0.4)	1 (25.0)	3 (75.0)	1 (25.0)	3 (75.0)
Living with unrelated roommates					
No	901 (81.4)	93 (12.7)	639 (87.3)	156 (18.1)^c^	704 (81.9)
Yes	206 (18.6)	25 (14.0)	154 (86.0)	45 (22.7)	153 (77.3)
Living with other farmworkers					
No	281 (25.4)	26 (11.6)	198 (88.4)	49 (18.4)	218 (81.6)
Yes	823 (74.3)	92 (13.5)	592 (86.5)	151 (19.2)	637 (80.8)
No answer	3 (0.3)	0	3 (100.0)	1 (33.3)	2 (66.7)
Persons per bedroom					
≤2	703 (63.5)	71 (12.3)	505 (87.7)	113 (17.0)^b^	553 (83.0)
>2	404 (36.5)	47 (14.0)	288 (86.0)	88 (22.4)	304 (77.6)
Access to washing machine at home					
No	411 (37.1)	49 (14.2)	296 (85.8)	77 (19.5)	318 (80.5)
Yes	696 (62.9)	69 (12.2)	497 (87.8)	124 (18.7)	539 (81.3)
Left home for nonessential reasons during past 2 wk					
No	957 (86.5)	104 (13.0)	693 (87.0)	169 (18.5)	745 (81.5)
Yes	144 (13.0)	12 (11.0)	97 (89.0)	29 (21.0)	109 (79.0)
No answer	6 (0.5)	2 (40.0)	3 (60.0)	3 (50.0)	3 (50.0)
Used public transportation or ride share services in past 2 wk					
No	1039 (93.9)	113 (13.2)^c^	745 (86.8)	189 (19.1)	801 (80.9)
Yes	62 (5.6)	3 (6.3)	45 (93.8)	8 (14.5)	53 (85.5)
No answer	6 (0.5)	2 (40.0)	3 (60.0)	3 (50.0)	3 (50.0)
Attended social gatherings with nonhousehold members in past 2 wk					
No	993 (89.7)	105 (12.8)	714 (87.2)	181 (19.1)	768 (80.9)
Yes	112 (10.1)	13 (14.4)	77 (85.6)	20 (18.5)	88 (81.5)
No answer	2 (0.2)	0	2 (100.0)	0	1 (100.0)
Attended indoor gatherings with nonhousehold members in past 2 wk					
No	1046 (94.5)	109 (12.6)^c^	753 (87.4)	192 (19.2)	807 (80.8)
Yes	59 (5.3)	9 (19.1)	38 (80.9)	9 (15.5)	49 (84.5)
No answer	2 (0.2)	0	2 (100.0)	0	1 (100.0)
Face covering use while <6 ft away from others all of the time					
No	85 (7.7)	4 (5.4)^b^	70 (94.6)	13 (16.0)	68 (84.0)
Yes	1022 (92.3)	114 (12.6)	723 (86.4)	188 (19.2)	789 (80.8)
Hand washing when returning home or after touching something all or most of the time					
No	33 (3.0)	3 (11.1)	24 (88.9)	5 (16.1)	26 (83.9)
Yes	1074 (97.0)	115 (13.0)	769 (87.0)	196 (19.1)	831 (80.9)
Possible exposure to someone with COVID-19 at home in the past 2 wk^d^					
No	986 (89.1)	79 (9.8)^b^	725 (90.2)	178 (18.8)	767 (81.2)
Yes	121 (10.9)	39 (36.4)	68 (63.6)	23 (20.4)	90 (79.6)
Possible exposure to someone with COVID-19 at home since the start of the pandemic^e^					
No	909 (82.1)	68 (9.2)^b^	675 (90.9)	152 (17.4)^b^	723 (82.6)
Yes	198 (17.9)	50 (29.8)	118 (70.2)	49 (26.8)	134 (73.2)

Abbreviations: IgG, immunoglobulin G; TMA, transcription-mediated amplification.

^a^ Missing entries were excluded from bivariate analyses.

^b^ *P* < .05.

^c^ *P* < .20.

^d^ Lived with someone who had COVID-19 symptoms or positive in the past 2 weeks.

^e^ Lived with someone who had COVID-19 symptoms or positive since the start of the pandemic.

**Table 3.** Work-Related Risk Factors for TMA and IgG Positivity Among Farmworkers, Monterey County, 2020

Attribute	Individuals, No. (%)^a^				
	All enrolled (N = 1107)	SARS-CoV-2 infection			
		TMA positive (n = 911)		IgG positive (n = 1058)	
		Yes (n = 118)	No (n = 793)	Yes (n = 201)	No (n = 857)
H2A visa holder					
No	1029 (93.0)	107 (12.7)	733 (87.3)	188 (19.2)	792 (80.8)
Yes	65 (5.9)	9 (15.0)	51 (85.0)	11 (16.9)	54 (83.1)
No answer	13 (1.2)	2 (18.2)	9 (81.8)	2 (15.4)	11 (84.6)
Supervisor or mayordomo					
No	1015 (91.7)	111 (12.8)	756 (87.2)	183 (18.9)	784 (81.1)
Yes	50 (4.5)	7 (15.9)	37 (84.1)	9 (18.4)	40 (81.6)
No answer	42 (3.8)	0	0	9 (21.4)	33 (78.6)
Type of agricultural work ever or in the past 2 wk^b^					
Worked in the fields	825 (74.5)	100 (14.7)^c^	580 (85.3)	162 (20.4)^c^	633 (79.6)
Packing shed	133 (12.0)	11 (10.5)	94 (89.5)	21 (16.4)	107 (83.6)
Processing facility	63 (5.7)	4 (7.0)^d^	53 (93.0)	7 (12.1)^d^	51 (87.9)
Nursery	38 (3.4)	4 (12.1)	29 (87.9)	4 (11.4)	31 (88.6)
Truck driver	38 (3.4)	4 (12.1)	29 (87.9)	3 (9.1)^d^	30 (90.9)
Packing truck	22 (2.0)	1 (4.8)	20 (95.2)	2 (9.5)	19 (90.5)
Other	21 (1.9)	1 (5.3)	18 (94.7)	2 (10.5)	17 (89.5)
No answer	10 (0.9)	0	1 (100.0)	2 (20.0)	8 (80.0)
Worked indoors					
No	844 (76.2)	98 (14.3)^c^	589 (85.7)	166 (20.4)^c^	646 (79.6)
Yes	262 (23.7)	20 (9.0)	203 (91.0)	35 (14.3)	210 (85.7)
No answer	1 (0.1)	0	1 (100.0)	0	1 (100.0)
Crops worked ever or in the past 2 wk^b^					
Berries	236 (28.6)	14 (7.2)^c^	181 (92.8)	39 (16.7)^d^	194 (83.3)
Leafy greens	218 (26.4)	31 (17.9)^d^	142 (82.1)	50 (24.2)^d^	157 (75.8)
Broccoli	155 (18.8)	28 (18.9)^d^	120 (81.1)	24 (16.3)^d^	123 (83.7)
Grapes	59 (7.2)	10 (21.3)^d^	37 (78.7)	15 (26.3)	42 (73.7)
Peas	52 (6.3)	16 (30.8)^c^	36 (69.2)	5 (10.0)^e^	45 (90.0)
Cauliflower	42 (5.1)	5 (13.5)	32 (86.5)	11 (30.6)^d^	25 (69.4)
Celery	19 (2.3)	2 (11.8)	15 (88.2)	3 (18.8)	13 (81.3)
Artichokes	6 (0.7)	0	5 (100.0)	1 (16.7)	5 (83.3)
Other	158 (19.2)	9 (8.4)^c^	98 (91.6)	35 (22.6)	120 (77.4)
Commuted to work with nonhousehold members					
No	707 (63.9)	65 (10.9)^c^	530 (89.1)	124 (18.5)	548 (81.5)
Yes	380 (34.3)	53 (16.8)	263 (83.2)	72 (19.7)	294 (80.3)
No answer	20 (1.8)	0	0	5 (25.0)	15 (75.0)
Used face covering at work all the time					
No	112 (10.1)	11 (11.7)	83 (88.3)	19 (18.1)	86 (81.9)
Yes	992 (89.6)	107 (13.1)	709 (86.9)	182 (19.2)	768 (80.8)
No answer	3 (0.3)	0	1 (100.0)	0	3 (100.0)
Came within 6 ft from others while working					
No	501 (45.3)	52 (12.6)	361 (87.4)	95 (19.7)	388 (80.3)
Yes	568 (51.3)	64 (13.4)	413 (86.6)	97 (18.0)	441 (82.0)
No answer	38 (3.4)	2 (9.5)	19 (90.5)	9 (24.3)	28 (75.7)
Possible exposure to someone with COVID-19 at work in past 2 wk^f^					
No	958 (86.5)	83 (10.9)^c^	680 (89.1)	178 (19.3)	744 (80.7)
Yes	149 (13.5)	35 (23.6)	113 (76.4)	23 (16.9)	113 (83.1)
No answer	0	0	0	0	0
Possible exposure to someone with COVID-19 at work since the start of the pandemic^g^					
No	665 (60.1)	52 (9.9)^c^	471 (90.1)	117 (18.3)	524 (81.7)
Yes	442 (39.9)	66 (17.0)	322 (83.0)	84 (20.1)	333 (79.9)
No answer	0	0	0	0	0

Abbreviations: IgG, immunoglobulin G; TMA, transcription-mediated amplification.

^a^ Missing entries were excluded from bivariate analyses.

^b^ Bivariate analyses compared each agricultural job with all other jobs and working in each crop with working in all other crops. Some participants worked in a variety of jobs and crops.

^c^ *P* < .05.

^d^ *P* < .20.

^e^ *P* < .10.

^f^ Worked with someone who had COVID-19 symptoms, tested positive for SARS-CoV-2, or who quarantined in the past 2 weeks.

^g^ Worked with someone who had COVID-19 symptoms, tested positive for SARS-CoV-2, or who quarantined since the start of the pandemic.

**Table 4.** Employer-Provided Preventive Measures and Their Association With TMA and IgG Positivity Among Farmworkers, Monterey County, 2020

Attribute	Individuals, No. (%)^a^				
	All enrolled (N = 1107)	SARS-CoV-2 infection			
		TMA positive (n = 911)		IgG positive (n = 1058)	
		Yes (n = 118)	No (n = 793)	Yes (n = 201)	No (n = 857)
**Fever and symptoms screening upon arrival at workplace**					
Neither	495 (44.7)	54 (16.6)^b^	272 (83.4)	92 (19.2)	388 (80.8)
Either or both	611 (55.2)	64 (10.9)	521 (89.1)	109 (18.9)	468 (81.1)
No answer	1 (0.1)	0	0	0	1 (100.0)
**Employer provided face coverings**					
No	168 (15.2)	15 (12.1)	109 (87.9)	26 (16.0)	136 (84.0)
Yes	932 (84.2)	102 (13.0)	681 (87.0)	174 (19.6)	715 (80.4)
No answer	7 (0.6)	1 (25.0)	3 (75.0)	1 (14.3)	6 (85.7)
**Employer provided gloves**					
No	161 (14.5)	12 (9.0)^c^	121 (91.0)	26 (17.1)	126 (82.9)
Yes	945 (85.4)	106 (13.6)	671 (86.4)	175 (19.3)	730 (80.7)
No answer	1 (0.1)	0	1 (100.0)	0	1 (100.0)
**Employer provided eye shields**					
No	542 (49.0)	52 (11.7)	392 (88.3)	94 (18.1)	424 (81.9)
Yes	564 (50.9)	66 (14.2)	400 (85.8)	107 (19.9)	432 (80.1)
No answer	1 (0.1)	0	1 (100.0)	0	1 (100.0)
**Employer provided hand washing stations**					
No	6 (0.5)	1 (20.0)	4 (80.0)	2 (33.3)	4 (66.7)
Yes	1100 (99.4)	117 (12.9)	788 (87.1)	199 (18.9)	852 (81.1)
No answer	1 (0.1)	0	1 (100.0)	0	1 (100.0)
**Employer provided liquid soap and paper towels**					
No	15 (1.4)	2 (16.7)	10 (83.3)	4 (26.7)	11 (73.3)
Yes	1090 (98.5)	116 (12.9)	781 (87.1)	196 (18.8)	845 (81.2)
No answer	2 (0.2)	0	2 (100.0)	1 (50.0)	1 (50.0)
**Employer provided hand sanitizer**					
No	95 (8.6)	8 (11.4)	62 (88.6)	20 (22.5)	69 (77.5)
Yes	1011 (91.3)	110 (13.1)	730 (86.9)	181 (18.7)	787 (81.3)
No answer	1 (0.1)	0	1 (100.0)	0	1 (100.0)
**Workplace surfaces and tools regularly disinfected and kept clean**					
No	122 (11.0)	12 (12.8)	82 (87.2)	17 (14.8)	98 (85.2)
Yes	946 (85.5)	98 (12.5)	687 (87.5)	175 (19.3)	730 (80.7)
No answer	39 (3.5)	8 (25.0)	24 (75.0)	9 (23.7)	29 (76.3)
**Employer staggered breaks to reduce exposure**					
No	608 (54.9)	66 (13.4)	428 (86.6)	112 (19.2)	471 (80.8)
Yes	492 (44.4)	51 (12.3)	362 (87.7)	89 (19.0)	380 (81.0)
No answer	7 (0.6)	1 (25.0)	3 (75.0)	0	6 (100.0)
**Employer provided information on COVID-19 symptoms**					
No	64 (5.8)	6 (15.0)	34 (85.0)	17 (27.4)^d^	45 (72.6)
Yes	1040 (93.9)	112 (12.9)	758 (87.1)	184 (18.5)	809 (81.5)
No answer	3 (0.3)	0	1 (100.0)	0	3 (100.0)
**Employer provided information on how workers can protect themselves at work**					
No	37 (3.3)	1 (4.8)	20 (95.2)	12 (34.3)^b^	23 (65.7)
Yes	1067 (96.4)	117 (13.2)	772 (86.8)	189 (18.5)	831 (81.5)
No answer	3 (0.3)	0	1 (100.0)	0	3 (100.0)
**Employer provided information on how workers can protect themselves at home and in the community**					
No	70 (6.3)	3 (6.4)^c^	44 (93.6)	17 (25.4)^c^	50 (74.6)
Yes	1034 (93.4)	115 (13.3)	748 (86.7)	184 (18.6)	804 (81.4)
No answer	3 (0.3)	0	1 (100.0)	0	3 (100.0)
**Employer provided information on whom to call if workers were sick**					
No	134 (12.1)	18 (18.2)^c^	81 (81.8)	27 (20.9)	102 (79.1)
Yes	970 (87.6)	100 (12.3)	711 (87.7)	174 (18.8)	752 (81.2)
No answer	3 (0.3)	0	1 (100.0)	0	3 (100.0)
**Employer provided information on workers’ ability to get free testing and treatment if they were sick**					
No	303 (27.4)	31 (13.1)	206 (86.9)	62 (21.5)	227 (78.5)
Yes	800 (72.3)	87 (12.9)	585 (87.1)	138 (18.0)	627 (82.0)
No answer	4 (0.4)	0	2 (100.0)	1 (25.0)	3 (75.0)
**Employer provided information on where workers could get housing if they needed to quarantine or isolate away from home**					
No	607 (54.8)	70 (14.1)	428 (85.9)	112 (19.3)	467 (80.7)
Yes	496 (44.8)	48 (11.7)	363 (88.3)	88 (18.5)	387 (81.5)
No answer	4 (0.4)	0	2 (100.0)	1 (25.0)	3 (75.0)
**Employer provided information on the importance of staying away from work if workers were sick**					
No	78 (7.0)	5 (9.1)	50 (90.9)	15 (19.5)	62 (80.5)
Yes	1024 (92.5)	113 (13.2)	741 (86.8)	185 (19.0)	791 (81.0)
No answer	5 (0.5)	0	2 (100.0)	1 (20.0)	4 (80.0)
**Employer provided information on workers’ benefit to get paid to stay away from work if they were sick**					
No	334 (30.2)	36 (13.7)	227 (86.3)	58 (18.4)	258 (81.6)
Yes	769 (69.5)	82 (12.7)	564 (87.3)	142 (19.2)	596 (80.8)
No answer	4 (0.4)	0	2 (100.0)	1 (25.0)	3 (75.0)
**Received education about COVID-19 from medical staff at workplace**					
No	714 (64.5)	76 (13.0)	508 (87.0)	126 (18.5)	556 (81.5)
Yes	373 (33.7)	41 (13.1)	271 (86.9)	73 (20.5)	283 (79.5)
No answer	20 (1.8)	1 (6.7)	14 (93.3)	2 (10.0)	18 (90.0)

Abbreviations: IgG, immunoglobulin G; TMA, transcription-mediated amplification.

^a^ Missing entries were excluded from bivariate analyses.

^b^ *P* < .05.

^c^ *P* < .20.

^d^ *P* < .10.

We used multiple imputation with chained equations to account for missing values (<2.5% missing for all variables) in our multivariable analyses. To account for differences between those recruited at clinics vs community events, as well as changes in the background positivity rate in Monterey County over the course of the study period,^2^ we grouped participants into strata by recruitment site and period (ie, July 16 to August 31, September 1 to 30, October 1 to 31, and November 1 to 30). We used conditional fixed-effects Poisson models^15^ to estimate adjusted relative risks (aRRs) while accounting for differences among strata, estimating robust standard errors using the Huber-White estimator. For multivariable models, statistical significance was set at *P* < .05. Analyses were conducted with Stata version 15.0 (StataCorp) and R version 3.6.1 (R Project for Statistical Computing). Given the limitations of relying on thresholds of statistical significance,^16^ we interpret our effect estimates based on their magnitude and precision, in light of the available sample size, instead of conditioning all conclusions on binary significance testing.

## Results

Of 1107 participants (581 [52.5%] women), 922 (83.3%) were born in Mexico, 488 (44.1%) had primary school or lower levels of educational attainment, 697 (63.0%) were married or living as married, and 881 (79.6%) had overweight or obesity (defined as body mass index [BMI; calculated as weight in kilograms divided by height in meters squared] ≥25.0) (Table 1). Participants had a mean (SD) age of 39.7 (12.6) and had lived in the United States for a mean (SD) of 21.3 (11.1) years. Overall, 940 (84.9%) spoke Spanish at home, and 110 (9.9%) spoke 1 of 11 Indigenous languages (eg, Mixteco, Zapoteco, and Triqui). Half of participants (557 [50.3%]) reported household earnings of less than $25 000 per year. Approximately three-quarters (829 [74.9%]) lived with children, including 408 (36.9%) who lived with children aged 5 years or younger, and 206 (18.6%) lived with unrelated roommates (Table 2). Farmworkers lived with a mean (SD) of 5.5 (2.5) household members, and 404 (36.5%) lived in crowded conditions (ie, >2 persons/bedroom). Overall, 198 (17.9%) reported living with someone who had symptoms of COVID-19 or were known to be infected with SARS-CoV-2 since the pandemic started, and 121 (10.9%) reported such exposures at home in the 2 weeks preceding their test.

A total of 825 participants (74.5%) worked in the fields and farmed a variety of crops; the most common were berries (236 [28.6%]), leafy greens (218 [26.4%]), and broccoli (155 [18.8%]) (Table 3). Overall, 992 farmworkers (89.6%) reported using a face covering at work, and 380 (34.3%) commuted to work with members of other households. Nearly 40% (442 [39.9%]) worked with someone who had symptoms of COVID-19 or who was known to be infected with SARS-CoV-2 during the pandemic, and 149 (13.5%) reported such workplace exposure during the 2 weeks preceding their testing date. Almost all farmworkers reported that their employers provided them with hand sanitizer, gloves, face coverings, and handwashing stations; disinfected surfaces and tools regularly; and provided them with information on how to prevent SARS-CoV-2 transmission at work (Table 4). However, 495 (44.7%) reported that their employer did not screen for fever and symptoms on arrival at the workplace, which was recommended as part of a countywide agricultural advisory.^17^

### Risk Factors for Positive SARS-CoV-2 Infection Results on TMA Tests

A total of 118 of the 911 participants (13.0%) who worked in agriculture in the 2 weeks preceding enrollment had positive results for SARS-CoV-2 infection on their TMA test, including 95 (18.4%) recruited at the clinics and 23 (5.8%) recruited via outreach (Table 1).^2^ Notably, we found that having a lower educational level, speaking Indigenous languages at home, living in the community of Greenfield, working in the fields, not working indoors, commuting to work with nonhousehold members, living or working with someone who had symptoms of COVID-19 or with known infection in the preceding 2 weeks, and not being screened for either fever or COVID-19 symptoms on arrival at work were factors associated with a higher prevalence of TMA-positive SARS-CoV-2 infection. We also observed correlations between some of these characteristics (eFigure in Supplement 1).

In multivariable analyses, the prevalence of positive results on TMA tests was higher among individuals who had only primary school or no education (aRR, 1.32; 95% CI, 0.99-1.76; non–statistically significant finding), spoke an Indigenous language at home (aRR, 1.30; 95% CI, 0.97-1.73; non–statistically significant finding), or lived with (aRR, 2.98; 95% CI, 2.06-4.32) or worked with (aRR, 1.59; 95% CI, 1.18-2.14) someone who had symptoms of COVID-19 or was known to be infected with SARS-CoV-2 in the previous 2 weeks (Figure, A). Additionally, working in the fields (vs agricultural work in all other settings) was associated with higher risk of a positive result on TMA testing (aRR, 1.60; 95% CI, 1.03-2.50). In contrast, farmworkers screened by employers for symptoms of COVID-19 or elevated temperature had a lower prevalence of TMA positivity (aRR, 0.79; 0.61-1.01; non–statistically significant finding) (Figure, A).

**Figure.**
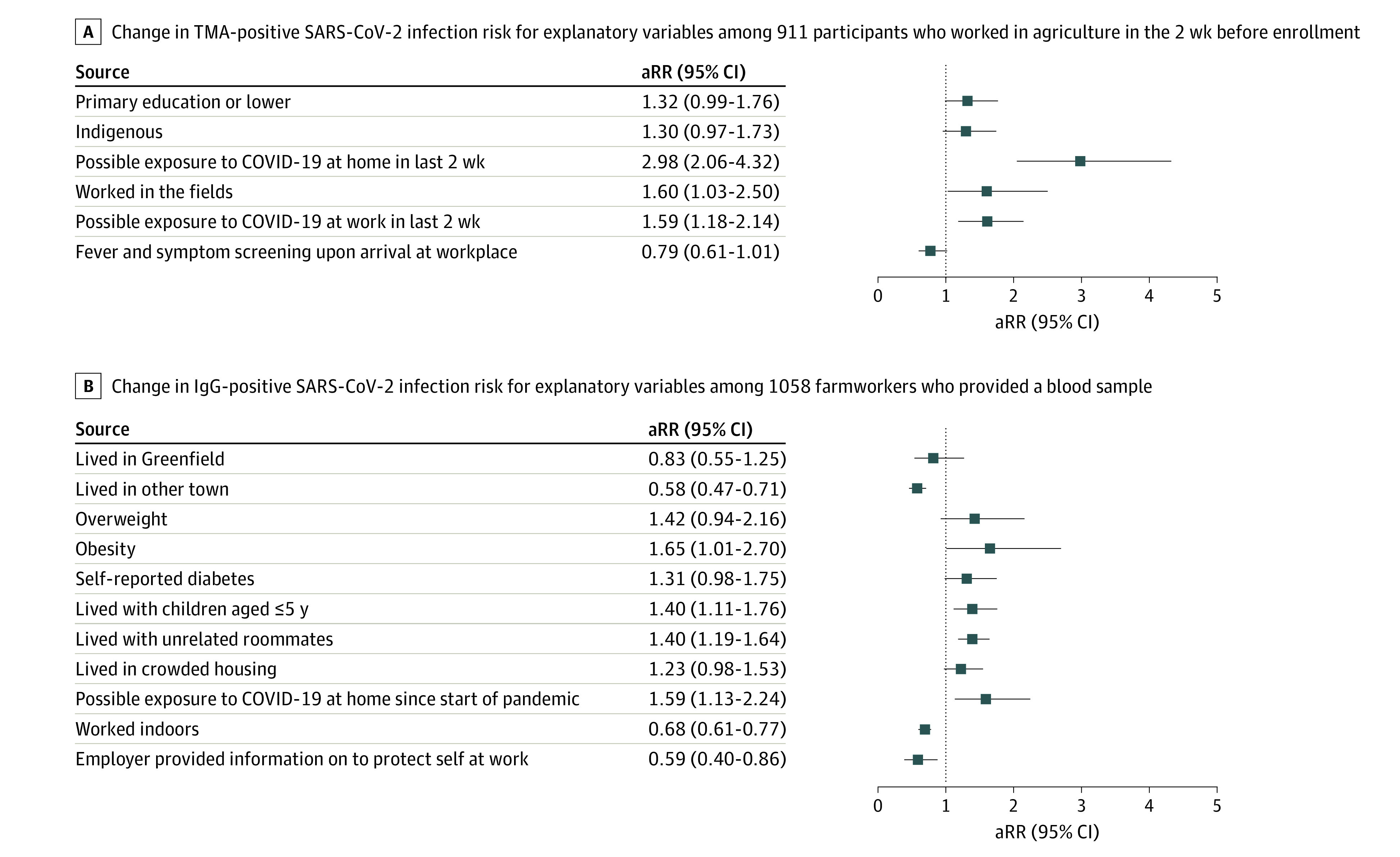
Risk Factors for SARS-CoV-2 Infection Figure shows adjusted relative risks (aRR) and 95% CIs from conditional fixed-effects Poisson models. IgG indicates immunoglobulin G; TMA, transcription-mediated amplification.

### Risk Factors for Positive SARS-CoV-2 Result on IgG Test

We found that 201 of the 1058 participants (19.0%) who provided a blood sample had a positive result for SARS-CoV-2 infection on their IgG test, with similar prevalence among those tested in the clinics (97 [18.4%]) and at community sites (104 [19.5%]) (Table 1).^2^ Among the 118 farmworkers who had positive results on their TMA test, 22 (18.6%) also had positive results on their IgG test (9 [7.6%] missing antibody status), whereas among the 793 participants who had negative results on their TMA test, 132 (16.7%) had positive results on their IgG test (40 [5.0%] missing antibody status). In bivariate analyses, we observed that having a lower educational level, living in Salinas or Greenfield (vs other towns), having overweight or obesity, living in large households or with children 5 years or younger, living in crowded housing, having ever lived with someone who had symptoms of COVID-19 or were known to be infected with SARS-CoV-2, and working in the fields were factors associated with a higher prevalence of IgG-positive SARS-CoV-2 infection (Table 1, Table 2, Table 3). We also found that working indoors and working for an employer who provided farmworkers with information on how to protect themselves at work were conditions associated with a lower prevalence of IgG positivity (Table 3 and Table 4).

In multivariable analyses, we found that participants who had obesity (ie, BMI ≥30; aRR, 1.65; 95% CI, 1.01-2.70), overweight (ie, BMI 25.0-29.9; aRR, 1.42; 95% CI, 0.94-2.16; non–statistically significant finding), or diabetes (aRR, 1.31; 95% CI, 0.98-1.75) had a higher prevalence of positive results on their IgG tests (Figure, B). We also identified a higher prevalence of IgG positivity among those living with children 5 years or younger (aRR, 1.40; 95% CI, 1.11-1.76), with unrelated roommates (aRR, 1.40; 95% CI, 1.19-1.64), or in crowded housing (aRR, 1.23; 95% CI, 0.98-1.53; non–statistically significant finding) and those who had ever lived with someone who had symptoms of COVID-19 or were known to be infected with SARS-CoV-2 (aRR, 1.59; 95% CI, 1.13-2.24). Farmworkers who lived outside the region’s largest communities of Salinas and Greenfield (aRR, 0.58; 95% CI, 0.47-0.71), worked indoors (aRR, 0.68; 95% CI, 0.61-0.77), or whose employer provided them with information on how to protect themselves at work (aRR, 0.59; 95% CI, 0.40-0.86) had a lower risk of having a positive result for SARS-CoV-2 infection on their IgG tests (Figure, B).

## Discussion

In this primarily Mexican-born and very low-income farmworker population in California, individuals with less than primary school–level education, who spoke an Indigenous language at home, who worked in the fields rather than elsewhere in agriculture, and were exposed to a known or suspected COVID-19 case at home or in the workplace had a higher prevalence of TMA-positive SARS-CoV-2 infection. We also found that IgG-positive SARS-CoV-2 infection was associated with outdoor work and with residential exposures (living with children, unrelated roommates, or an individual with known or suspected COVID-19). Those living in the more urban areas of the county were particularly at risk for IgG positivity, as were those who had obesity or diabetes. As evidence of the importance of health education, farmworkers who reported that their employer provided them with information on COVID-19 protection had a lower risk of IgG positivity for SARS-CoV-2 infection.

Our study suggests several routes of SARS-CoV-2 exposure that may be of importance to the farmworker population. Unsurprisingly, individuals living in crowded housing or with unrelated roommates had a higher prevalence of IgG positivity for SARS-CoV-2 infection. Independent of these findings, we also observed a higher IgG positivity prevalence among individuals living with children 5 years or younger. While the role of children in SARS-CoV-2 transmission has been uncertain in many populations, in part due to lower risk of symptoms and lower frequency of testing at younger ages,^18,19,20^ recent investigations have demonstrated equivalent viral load across ages^21^ and higher risk of transmission from infected children than from adults, given similar household exposures.^22^ While schools and formal daycare establishments were closed during our study, informal or home-based childcare arrangements with relatives or friends may have led to additional exposure to infection. Taken together, our findings suggest substantial risk of infection associated with residential exposures in this low-income population of essential workers.

Several workplace factors were also associated with infection risk. Farmworkers whose employers provided informational resources on preventing COVID-19 at work had 41% lower risk of IgG positivity for SARS-CoV-2 infection, whereas farmworkers whose employers screened them for symptoms or fever had a 21% lower risk of TMA positivity. This reduction could owe to benefits of health education, as well as more stringent efforts by employers to reduce risk by providing education and screenings. Individuals working outside and in the fields were more likely to have both TMA and IgG positivity. Whereas indoor exposures are thought to be associated with the greatest risk of transmission,^23^ a lower perceived sense of risk during outdoor work or socioeconomic differences between outdoor and indoor workers may contribute to the observed association in our study. While the estimated risk ratio for infection associated with workplace exposure was lower than that for household exposure, this difference could in part reflect misclassification if individuals are more likely to know about the health of household members. Previously, we have reported higher SARS-CoV-2 test positivity among farmworkers than among age- and sex-matched adults from the same communities who also received testing at CSVS,^2^ further supporting the hypothesis that workplace exposures specific to agriculture may be of importance to SARS-CoV-2 transmission.

Finally, we observed that farmworkers who spoke an Indigenous language at home and those with less than primary school–level education had a higher prevalence of positive results on their TMA tests at the time of enrollment. Those who spoke Indigenous languages also had a lower educational level and had more recently arrived in the United States. They lived in more crowded conditions and were more likely to live in Greenfield, work in the fields, and commute to work with non–household members (eFigure in Supplement 1). Only limited COVID-19 health messages have been provided in Indigenous languages, which are primarily not written languages.

We found associations of IgG positivity for SARS-CoV-2 infection with comorbid conditions. While it is known that obesity increases the risk of severe COVID-19 illness,^24^ we observed an increased risk of IgG positivity among individuals with obesity. This finding is consistent with a recent meta-analysis of 20 studies,^24^ which found 46% greater odds of SARS-CoV-2 infection among individuals with obesity, possibly related to alterations in systemic metabolism, including altered adipokines^25,26,27^ and chronic low-grade inflammation.^28,29^ Similarly, diabetes can attenuate the synthesis of proinflammatory cytokines and their downstream acute phase reactants,^30^ but also impair macrophage and lymphocyte functions.^31^ As obesity and diabetes are prevalent among farmworkers as well as other low-income Latino populations, our findings that these conditions are associated with higher risk of infection add to previous concerns based on the knowledge that these conditions may also exacerbate risk of adverse clinical outcomes.

### Strengths and Limitations

Our work represents one of the first epidemiological studies to address risk factors for SARS-CoV-2 infection among US farmworkers and substantiates earlier concerns^32,33,34,35^ that living and working conditions in this population may contribute to risk of infection. However, several limitations should be considered. We cannot determine how well our sample represents the farmworker population, many of whom are hidden due to their informal workforce participation and undocumented status.^36^ In addition, under the busy conditions of study recruitment in this clinical setting, we could not document participation rates systematically. As we excluded individuals who did not speak Spanish or English sufficiently well to participate, our study likely underrepresents Indigenous populations. We observed differences in prevalence of TMA positivity but not IgG positivity for SARS-CoV-2 infection between study participants recruited at clinics and those recruited via community outreach events,^2^ as individuals seeking testing at clinics were more likely to be symptomatic or to report recent known exposure; to mitigate confounding, we defined strata by recruitment site. Furthermore, waning antibodies, particularly for individuals experiencing mild or asymptomatic infection,^37^ may have contributed to misclassification for individuals infected early in the pandemic. Misclassification of active infections may have also occurred given that TMA tests can remain positive after a person has recovered from a SARS-CoV-2 infection due to detection of nonviable viral RNA.^38^ Additionally, many identified risk factors were highly correlated, making it difficult to separate their unique associations. Larger studies of farmworkers that allow for fine-grained analysis of living and working conditions with SARS-CoV-2 transmission are warranted.

## Conclusions

Our findings underscore the urgent need to intervene on modifiable risk factors associated with SARS-CoV-2 infection among farmworkers in California, such as increasing availability of isolation facilities to reduce exposure to COVID-19 cases at home and access to paid medical leave to avoid transmission in the workplace. Individuals who spoke Indigenous languages at home, had lower levels of formal education, and lived in rural communities had a higher prevalence of infection in our study, which demonstrate disparities even within this very low-income population. Efficacious vaccines should be distributed to farmworkers with urgency owing to the high risk of infection in this population and the essential nature of their work.

## Notes

Supplement 1. **eTable 1.** Sociodemographic and Health-Related Risk Factors for TMA and IgG Positivity Among Farmworkers, Monterey County, 2020 **eTable 2.** Household and Community Risk Factors for TMA and IgG Positivity Among Farmworkers, Monterey County, 2020 **eTable 3.** Work-Related Risk Factors for TMA and IgG Positivity Among Farmworkers, Monterey County, 2020 **eTable 4.** Employer-Provided Preventive Measures and Their Association With TMA and IgG Positivity Among Farmworkers, Monterey County, 2020 **eFigure.** Correlation Heat Map of Risk Factors Associated With TMA and IgG Positivity Among Farmworkers, Monterey County, 2020

Supplement 2.
Nonauthor Collaborators
