# Supplementary material for: Risk Factors Associated With SARS-CoV-2 Infection Among Farmworkers in Monterey County, California
Source: JAMA Netw Open. 2021 Sep 15;4(9):e2124116. doi: 10.1001/jamanetworkopen.2021.24116 (PMC8444020; doi:10.1001/jamanetworkopen.2021.24116)
Supplement: Supplement 1. — eTable 1. Sociodemographic and Health-Related Risk Factors for TMA and IgG Positivity Among Farmworkers, Monterey County, 2020 eTable 2. Household and Community Risk Factors for TMA and IgG Positivity Among Farmworkers, Monterey County, 2020 eTable 3. Work-Related Risk Factors for TMA and IgG Positivity Among Farmworkers, Monterey County, 2020 eTable 4. Employer-Provided Preventive Measures and Their Association With TMA and IgG Positivity Among Farmworkers, Monterey County, 2020 eFigure. Correlation Heat Map of Risk Factors Associated With TMA and IgG Positivity Among Farmworkers, Monterey County, 2020 [file jamanetwopen-e2124116-s001.pdf]

Supplemental Online Content

Mora AM, Lewnard JA, Kogut K, et al; for the CHAMACOS-Project-19 Study Team. Risk factors associated with SARS-CoV-2 infection among farmworkers in Monterey County, California. *JAMA Netw Open.* 2021;4(9):e2124116. doi:10.1001/jamanetworkopen.2021.24116

eTable 1. Sociodemographic and Health-Related Risk Factors for TMA and IgG Positivity Among Farmworkers, Monterey County, 2020

eTable 2. Household and Community Risk Factors for TMA and IgG Positivity Among Farmworkers, Monterey County, 2020

eTable 3. Work-Related Risk Factors for TMA and IgG Positivity Among Farmworkers, Monterey County, 2020

eTable 4. Employer-Provided Preventive Measures and Their Association With TMA and IgG Positivity Among Farmworkers, Monterey County, 2020

eFigure. Correlation Heat Map of Risk Factors Associated With TMA and IgG Positivity Among Farmworkers, Monterey County, 2020

This supplemental material has been provided by the authors to give readers additional information about their work.

© 2021 Mora AM et al. *JAMA Network Open.*

eTable 1. Sociodemographic and Health-Related Risk Factors for TMA and IgG Positivity Among Farmworkers, Monterey County, 2020

Attribute	TMA-positive SARS-CoV-2 infection			IgG-positive SARS-CoV-2 infection		
	**All enrolled** N=911	**Yes** N=118	**No** N=793	**All enrolled** N=1058	**Yes** N=201	**No** N=857
	n (%) or M ± SD	*n* (%) or M ± SD	*n* (%) or M ± SD	*n* (%) or M ± SD	*n* (%) or M ± SD	*n* (%) or M ± SD
Recruitment site						
Clinics	515 (56.5)	95 (80.5)	420 (53.0)	526 (49.7)	97 (48.3)	429 (50.1)
Community outreach	396 (43.5)	23 (19.5)	373 (47.0)	532 (50.3)	104 (51.7)	428 (49.9)
Agricultural work in the preceding two weeks						
No	--	--	--	193 (18.2)	45 (22.4)	148 (17.3)
Yes	911 (100.0)	118 (100.0)	793 (100.0)	865 (81.8)	156 (77.6)	709 (82.7)
Sex						
Female	460 (50.5)	60 (50.8)	393 (49.6)	547 (51.7)	99 (49.3)	448 (52.3)
Male	451 (49.5)	58 (49.2)	400 (50.4)	511 (48.3)	102 (50.7)	409 (47.7)
Age (years)	39.6 ± 12.2	39.6 ± 11.0	39.6 ± 12.4	39.6 ± 12.6	39.6 ± 12.3	39.6 ±12.6
18-29	225 (24.7)	27 (22.9)	198 (25.0)	263 (24.9)	43 (21.4)	220 (25.7)
30-39	224 (24.6)	29 (24.6)	195 (24.6)	262 (24.8)	59 (29.4)	203 (23.7)
40-49	256 (28.1)	42 (35.6)	214 (27.0)	284 (26.8)	59 (29.4)	225 (26.3)
50-59	157 (17.2)	16 (13.6)	141 (17.8)	186 (17.6)	27 (13.4)	159 (18.6)
≥60	49 (5.4)	4 (3.4)	45 (5.7)	63 (6.0)	13 (6.5)	50 (5.8)
Education						
Primary school complete or less	409 (44.9)	63 (53.6)	346 (43.6)	472 (44.6)	100 (49.8)	372 (43.4)
More than primary school	501 (55.0)	55 (46.6)	446 (56.2)	585 (55.3)	101(50.2)	484 (56.5)
No answer	1 (0.1)	0 (0.0)	1 (0.1)	1 (0.1)	0 (0.0)	1 (0.1)
Marital status						
Not married or living as married	327 (35.9)	50 (42.4)	277 (34.9)	388 (36.7)	69 (34.3)	319 (37.2)
Married or living as married	583 (64.0)	67 (56.8)	516 (65.1)	669 (63.2)	132 (65.7)	537 (62.7)
No answer	1 (0.1)	1 (0.8)	0 (0.0)	1 (0.1)	0 (0.0)	1 (0.1)
Annual household income						
<$25,000	456 (50.1)	66 (55.9)	390 (49.2)	536 (50.7)	101 (50.2)	435 (50.8)
≥$25,000	415 (45.6)	48 (40.7)	367 (46.3)	466 (44.0)	86 (42.8)	380 (44.3)
No answer	40 (4.4)	4 (3.4)	36 (4.5)	56 (5.3)	14 (7.0)	42 (4.9)
Language spoken at home						
Spanish	772 (84.7)	96 (81.4)	676 (85.2)	896 (84.7)	166 (82.6)	730 (85.2)
English	42 (4.6)	0 (0.0)	42 (5.3)	55 (5.2)	12 (6.0)	43 (5.0)
Indigenous	97 (10.7)	22 (18.6)	75 (9.5)	107 (10.1)	23 (11.4)	84 (9.8)
No answer	0 (0.0)	0 (0.0)	0 (0.0)	0 (0.0)	0 (0.0)	0 (0.0)

© 2021 Mora AM et al. *JAMA Network Open.*

Country of birth						
Mexico	773 (84.9)	104 (88.1)	669 (84.4)	884 (83.6)	163 (81.1)	721 (84.1)
US	100 (11.0)	7 (5.9)	93 (11.7)	135 (12.8)	31 (15.4)	104 (12.1)
Other	38 (4.2)	7 (5.9)	31 (3.9)	39 (3.7)	7 (3.5)	32 (3.7)
Years in US	20.9 ± 11.1	20.2 ± 10.9	21.0 ± 11.2	21.3 ± 11.1	21.3 ± 10.6	21.2 ± 11.3
<15	231 (25.4)	38 (32.2)	193 (24.3)	255 (24.1)	46 (22.9)	209 (24.4)
15-19	164 (18.0)	17 (14.4)	147 (18.5)	183 (17.3)	44 (21.9)	139 (16.2)
20-29	252 (27.7)	34 (28.8)	218 (27.5)	281 (26.6)	49 (24.4)	232 (27.1)
≥30	164 (18.0)	22 (18.6)	142 (17.9)	203 (19.2)	31 (15.4)	172 (20.1)
Entire life	100 (11.0)	7 (5.9)	93 (11.7)	135 (12.8)	31 (15.4)	104 (12.1)
No answer	0 (0.0)	0 (0.0)	0 (0.0)	1 (0.1)	0 (0.0)	1 (0.1)
Community of residence						
Salinas	383 (42.0)	40 (33.9)	343 (43.3)	468 (44.2)	99 (49.3)	369 (34.1)
Greenfield	283 (31.1)	56 (47.5)	227 (28.6)	297 (28.1)	63 (31.3)	234 (27.3)
Other town	245 (26.9)	22 (18.6)	223 (28.1)	293 (27.7)	39 (19.4)	254 (29.6)
Smoking						
Never smoked	729 (80.0)	99 (83.9)	630 (79.5)	855 (80.8)	157 (78.1)	698 (81.4)
Former smoker	140 (15.4)	16 (13.6)	124 (15.6)	154 (14.6)	36 (17.9)	118 (13.8)
Current smoker	41 (4.5)	3 (2.5)	38 (4.8)	48 (4.5)	8 (4.0)	40 (4.7)
No answer	1 (0.1)	0 (0.0)	1 (0.1)	1 (0.1)	0 (0.0)	1 (0.1)
Body mass index (measured)	29.6 ± 5.5	29.2 ± 4.7	29.7 ± 5.6	29.6 ± 5.5	30.4 ± 5.4	29.4 ± 5.5
<25 (underweight or normal)	167 (18.3)	18 (15.3)	149 (18.8)	192 (18.1)	24 (11.9)	168 (19.6)
25-29.9 (overweight)	347 (38.1)	45 (38.1)	302 (38.1)	404 (38.2)	75 (37.3)	329 (38.4)
≥30 (obese)	375 (41.2)	49 (41.5)	326 (41.1)	437 (41.3)	95 (47.3)	342 (39.9)
Not collected	22 (2.4)	6 (5.1)	16 (2.0)	25 (2.4)	7 (3.5)	18 (2.1)
Self-reported hypertension						
No	786 (86.3)	106 (89.8)	680 (85.8)	915 (86.5)	171 (85.1)	744 (86.8)
Yes	122 (13.4)	12 (10.2)	110 (13.9)	139 (13.1)	29 (14.4)	110 (12.8)
No answer	3 (0.3)	0 (0.0)	3 (0.4)	4 (0.4)	1 (0.5)	3 (0.4)
Self-reported diabetes						
No	803 (88.1)	109 (92.4)	694 (87.5)	934 (88.3)	172 (85.6)	762 (88.9)
Yes	105 (11.5)	9 (7.6)	96 (12.1)	120 (11.3)	28 (13.9)	92 (10.7)
No answer	3 (0.3)	0 (0.0)	3 (0.4)	4 (0.4)	1 (0.5)	3 (0.4)

© 2021 Mora AM et al. *JAMA Network Open.*

eTable 2. Household and Community Risk Factors for TMA and IgG Positivity Among Farmworkers, Monterey County, 2020

Attribute	TMA-positive SARS-CoV-2 infection			IgG-positive SARS-CoV-2 infection		
	**All enrolled** N=911	**Yes** N=118	**No** N=793	**All enrolled** N=1058	**Yes** N=201	**No** N=857
	n (%) or M ± SD	*n* (%) or M ± SD	*n* (%) or M ± SD	*n* (%) or M ± SD	*n* (%) or M ± SD	*n* (%) or M ± SD
Type of housing						
House	443 (48.6)	60 (50.8)	383 (48.3)	490 (46.3)	101 (50.2)	389 (45.4)
Apartment	372 (40.8)	48 (40.7)	324 (40.9)	468 (44.2)	85 (42.3)	383 (44.7)
Hotel or motel	36 (4.0)	6 (5.1)	30 (3.8)	37 (3.5)	5 (2.5)	32 (3.7)
Trailer or mobile home	39 (4.3)	3 (2.5)	36 (4.5)	41 (3.9)	8 (4.0)	33 (3.9)
Other	21 (2.3)	1 (0.8)	20 (2.5)	22 (2.1)	2 (1.0)	20 (2.3)
Household size	5.4 ± 2.3	5.5 ± 2.4	5.4 ± 2.3	5.5 ± 2.6	5.9 ± 2.6*	5.4 ± 2.6
0 others	11 (1.2)	2 (1.7)	9 (1.1)	12 (1.1)	3 (1.5)	9 (1.1)
1-3 others	331 (36.3)	41 (34.7)	290 (36.6)	379 (35.8)	58 (28.9)	321 (37.5)
4-6 others	414 (45.4)	51 (43.2)	363 (45.8)	486 (45.9)	93 (46.3)	393 (45.9)
≥7 others	155 (17.0)	24 (20.3)	131 (16.5)	181 (17.1)	47 (23.4)	134 (15.6)
Children <18 living in the home						
No	235 (25.8)	28 (23.7)	207 (26.1)	268 (25.3)	43 (21.4)	225 (26.3)
Yes	675 (74.1)	90 (76.3)	585 (73.8)	789 (74.6)	157 (78.1)	632 (73.7)
No answer	1 (0.1)	0 (0.0)	1 (0.1)	1 (0.1)	1 (0.5)	0 (0.0)
Children ≤5 living in the home						
No	577 (63.3)	79 (66.9)	498 (62.8)	669 (63.2)	110 (54.7)	559 (65.2)
Yes	334 (36.7)	39 (33.1)	295 (37.2)	389 (36.8)	91 (45.3)	298 (34.8)
Children attending school/daycare						
No	831 (91.2)	105 (89.0)	726 (91.6)	976 (92.2)	184 (91.5)	792 (92.4)
Yes	76 (8.3)	12 (10.2)	64 (8.1)	78 (7.4)	16 (8.0)	62 (7.2)
No answer	4 (0.4)	1 (0.8)	3 (0.4)	4 (0.4)	1 (0.5)	3 (0.4)
Living with unrelated roommates						
No	732 (80.4)	93 (78.8)	639 (80.6)	860 (81.3)	156 (77.6)	704 (82.1)
Yes	179 (19.6)	25 (21.2)	154 (19.4)	198 (18.7)	45 (22.4)	153 (17.9)
Living with other farmworkers						
No	224 (24.6)	26 (22.0)	198 (25.0)	267 (25.2)	49 (24.4)	218 (25.4)
Yes	684 (75.1)	92 (78.0)	592 (74.7)	788 (74.5)	151 (75.1)	637 (74.3)
No answer	3 (0.3)	0 (0.0)	3 (0.4)	3 (0.3)	1 (0.5)	2 (0.2)
Household crowding						
≤2 persons per bedroom	576 (63.2)	71 (60.2)	505 (63.7)	666 (62.9)	113 (56.2)	553 (64.5)
>2 persons per bedroom	335 (36.8)	47 (39.8)	288 (36.3)	392 (37.1)	88 (43.8)	304 (35.5)

© 2021 Mora AM et al. *JAMA Network Open.*

Access to washing machine at home						
No	345 (37.9)	49 (41.5)	296 (37.3)	395 (37.3)	77 (38.3)	318 (37.1)
Yes	566 (62.1)	69 (58.5)	497 (62.7)	663 (62.7)	124 (61.7)	539 (62.9)
Left home for non-essential reasons (past 2 weeks)						
No	797 (87.5)	104 (88.1)	693 (87.4)	914 (86.4)	169 (84.1)	745 (86.9)
Yes	109 (12.0)	12 (10.2)	97 (12.2)	138 (13.0)	29 (14.4)	109 (12.7)
No answer	5 (0.5)	2 (1.7)	3 (0.4)	6 (0.6)	3 (1.5)	3 (0.4)
Used public transportation/ride share services (past 2 weeks)						
No	858 (94.2)	113 (95.8)	745 (93.9)	990 (93.6)	189 (94.0)	801 (93.5)
Yes	48 (5.3)	3 (2.5)	45 (5.7)	62 (5.9)	9 (4.5)	53 (6.2)
No answer	5 (0.5)	2 (1.7)	3 (0.4)	6 (0.6)	3 (1.5)	3 (0.4)
Attended social gatherings with non-household members (past 2 weeks)						
No	819 (89.9)	105 (89.0)	714 (90.0)	949 (89.7)	181 (90.0)	768 (89.6)
Yes	90 (9.9)	13 (11.0)	77 (9.7)	108 (10.2)	20 (10.0)	88 (10.3)
No answer	2 (0.2)	0 (0.0)	2 (0.3)	1 (0.1)	0 (0.0)	1 (0.1)
Attended indoor gatherings with non-household members (past 2 weeks)						
No	862 (94.6)	109 (92.4)	753 (95.0)	999 (94.4)	192 (95.5)	807 (94.2)
Yes	47 (5.2)	9 (7.6)	38 (4.8)	58 (5.5)	9 (4.5)	49 (5.7)
No answer	2 (0.2)	0 (0.0)	2 (0.3)	1 (0.1)	0 (0.0)	1 (0.1)
Face covering use while less than 6 feet away from others all of the time						
No	74 (8.1)	4 (3.4)	70 (8.8)	81 (7.7)	13 (6.5)	68 (7.9)
Yes	837 (91.9)	114 (96.6)	723 (91.2)	977 (92.3)	188 (93.5)	789 (92.1)
Hand washing when returning home or after touching something all or most of the time						
No	27 (3.0)	3 (2.5)	24 (3.0)	31 (2.9)	5 (2.5)	26 (3.0)
Yes	884 (97.0)	115 (97.5)	769 (97.0)	1027 (97.1)	196 (97.5)	831 (97.0)
Possible exposure to someone with COVID-19 at home in the past 2 weeks[a]						
No	804 (88.3)	79 (66.9)	725 (91.4)	945 (89.3)	178 (88.6)	767 (89.5)

© 2021 Mora AM et al. *JAMA Network Open.*

Yes	107 (11.7)	39 (33.1)	68 (8.6)	113 (10.7)	23 (11.4)	90 (10.5)
Possible exposure to someone with COVID-19 at home since the start of the pandemic[b]						
No	743 (81.6)	68 (57.6)	675 (85.1)	875 (82.7)	152 (75.6)	723 (84.4)
Yes	168 (18.4)	50 (42.4)	118 (14.9)	183 (17.3)	49 (24.4)	134 (15.6)

[a]Lived with someone who had COVID-19 symptoms or positive in the past 2 weeks.
[b]Lived with someone who had COVID-19 symptoms or positive since the start of the pandemic.

© 2021 Mora AM et al. *JAMA Network Open.*

eTable 3. Work-Related Risk Factors for TMA and IgG Positivity Among Farmworkers, Monterey County, 2020

Attribute	TMA-positive SARS-CoV-2 infection			IgG-positive SARS-CoV-2 infection		
	**All enrolled** N=911	**Yes** N=118	**No** N=793	**All enrolled** N=1058	**Yes** N=201	**No** N=857
	n (%) or M ± SD	*n* (%) or M ± SD	*n* (%) or M ± SD	*n* (%) or M ± SD	*n* (%) or M ± SD	*n* (%) or M ± SD
H2A visa holder						
No	840 (92.2)	107 (90.7)	733 (92.4)	980 (92.6)	188 (93.5)	792 (92.4)
Yes	60 (6.6)	9 (7.6)	51 (6.4)	65 (6.1)	11 (5.5)	54 (6.3)
No answer	11 (1.2)	2 (1.7)	9 (1.1)	13 (1.2)	2 (1.0)	11 (1.3)
Supervisor or mayordomo						
No	867 (95.2)	111 (94.1)	756 (95.3)	967 (91.4)	183 (91.0)	784 (91.5)
Yes	44 (4.8)	7 (5.9)	37 (4.7)	49 (4.6)	9 (4.5)	40 (4.7)
No answer	0 (0.0)	0 (0.0)	0 (0.0)	42 (4.0)	9 (4.5)	33 (3.9)
Type of agricultural work (ever/in the past 2 weeks)[a]						
Working in the fields	680 (74.6)	100 (84.8)	580 (73.1)	795 (75.1)	162 (80.6)	633 (73.9)
Packing shed	105 (11.5)	11 (9.3)	94 (11.9)	128 (12.1)	21 (10.5)	107 (12.5)
Processing facility	57 (6.3)	4 (3.4)	53 (6.7)	58 (5.5)	7 (3.5)	51 (6.0)
Nursery	33 (3.6)	4 (3.4)	29 (3.7)	35 (3.3)	4 (2.0)	31 (3.6)
Truck driver	33 (3.6)	4 (3.4)	29 (3.7)	33 (3.1)	3 (1.5)	30 (3.5)
Packing truck	21 (2.3)	1 (0.9)	20 (2.5)	21 (2.0)	2 (1.0)	19 (2.2)
Other	19 (2.1)	1 (0.9)	18 (2.3)	19 (1.8)	2 (1.0)	17 (2.0)
No answer	1 (0.1)	0 (0.0)	1 (0.1)	10 (1.0)	2 (1.0)	8 (0.9)
Worked indoors						
No	687 (75.5)	98 (93.1)	589 (74.3)	812 (76.7)	166 (82.6)	646 (75.4)
Yes	223 (24.5)	20 (16.9)	203 (25.6)	245 (23.2)	35 (17.4)	210 (24.5)
No answer	1 (0.1)	0 (0.0)	1 (0.1)	1 (0.1)	0 (0.0)	1 (0.1)
Crops (ever/in the past 2 weeks)[a]						
Berries	195 (28.7)	14 (14.0)	181 (31.2)	233 (29.3)	39 (24.1)	194 (30.7)
Leafy greens	173 (25.4)	31 (31.0)	142 (24.5)	207 (26.0)	50 (30.9)	157 (24.8)
Broccoli	148 (21.8)	28 (28.0)	120 (20.7)	147 (18.5)	24 (14.8)	123 (19.4)
Grapes	47 (6.9)	10 (10.0)	37 (6.4)	57 (7.2)	15 (9.3)	42 (6.6)
Peas	52 (7.7)	16 (16.0)	36 (6.2)	50 (6.3)	5 (3.1)	45 (7.1)
Cauliflower	37 (5.4)	5 (5.0)	32 (5.5)	36 (4.5)	11 (6.8)	25 (4.0)
Celery	17 (2.5)	2 (2.0)	15 (2.6)	16 (2.0)	3 (1.9)	13 (2.1)
Artichokes	5 (0.7)	0 (0.0)	5 (0.9)	6 (0.8)	1 (0.6)	5 (0.8)
Other	107 (15.7)	9 (9.0)	98 (16.9)	155 (19.5)	35 (21.6)	120 (19.0)

© 2021 Mora AM et al. *JAMA Network Open.*

Commuted to work with non-household members						
No	595 (65.3)	65 (55.1)	530 (66.8)	672 (63.5)	124 (61.7)	548 (63.9)
Yes	316 (34.7)	53 (44.9)	263 (33.2)	366 (34.6)	72 (35.8)	294 (34.3)
No answer	0 (0.0)	0 (0.0)	0 (0.0)	20 (1.9)	5 (2.5)	15 (1.8)
Used face covering at work all of the time						
No	94 (10.3)	11 (9.3)	83 (10.5)	105 (9.9)	19 (9.5)	86 (10.0)
Yes	816 (89.6)	107 (90.7)	709 (89.4)	950 (89.8)	182 (90.5)	768 (89.6)
No answer	1 (0.1)	0 (0.0)	1 (0.1)	3 (0.3)	0 (0.0)	3 (0.4)
Came within 6 feet from others while working						
No	413 (45.3)	52 (44.1)	361 (45.5)	483 (45.7)	95 (47.3)	388 (45.3)
Yes	477 (52.4)	64 (54.2)	413 (52.1)	538 (50.9)	97 (48.3)	441 (51.5)
No answer	21 (2.3)	2 (1.7)	19 (2.4)	37 (3.5)	9 (4.5)	28 (3.3)
Possible exposure to someone with COVID-19 at work in the past 2 weeks[b]						
No	763 (83.8)	83 (70.3)	680 (85.8)	922 (87.1)	178 (88.6)	744 (86.8)
Yes	148 (16.2)	35 (29.7)	113 (14.2)	136 (12.9)	23 (11.4)	113 (13.2)
No answer	0 (0.0)	0 (0.0)	0 (0.0)	0 (0.0)	0 (0.0)	0 (0.0)
Possible exposure to someone with COVID-19 at work since the start of the pandemic[c]						
No	523 (57.4)	52 (44.1)	471 (59.4)	641 (60.6)	117 (58.2)	524 (61.1)
Yes	388 (42.6)	66 (55.9)	322 (40.6)	417 (39.4)	84 (41.8)	333 (38.9)
No answer	0 (0.0)	0 (0.0)	0 (0.0)	0 (0.0)	0 (0.0)	0 (0.0)

[a]Bivariate analyses compared each agricultural job with all other jobs and working in each crop with working in all other crops. We note that some participants worked in a variety of jobs and crops.
[b]Worked with someone who had COVID-19 symptoms, tested positive for SARS-CoV-2, or who quarantined in the past 2 weeks.
[c]Worked with someone who had COVID-19 symptoms, tested positive for SARS-CoV-2, or who quarantined since the start of the pandemic.

© 2021 Mora AM et al. *JAMA Network Open.*

eTable 4. Employer-Provided Preventive Measures and Their Association With TMA and IgG Positivity Among Farmworkers, Monterey County, 2020

Attribute	TMA-positive SARS-CoV-2 infection			IgG-positive SARS-CoV-2 infection		
	**All enrolled** N=911	**Yes** N=118	**No** N=793	**All enrolled** N=1058	**Yes** N=201	**No** N=857
	n (%) or M ± SD	*n* (%) or M ± SD	*n* (%) or M ± SD	*n* (%) or M ± SD	*n* (%) or M ± SD	*n* (%) or M ± SD
Fever and symptoms screening upon arrival at workplace						
Neither	326 (35.8)	54 (45.8)	272 (34.3)	480 (45.4)	92 (45.8)	388 (45.3)
Either or both	585 (64.2)	64 (54.2)	521 (65.7)	577 (54.5)	109 (54.2)	468 (54.6)
No answer	0 (0.0)	0 (0.0)	0 (0.0)	1 (0.1)	0 (0.0)	1 (0.1)
Employer provided face coverings						
No	124 (13.6)	15 (12.7)	109 (13.7)	162 (15.3)	26 (12.9)	136 (15.9)
Yes	783 (86.0)	102 (86.4)	681 (85.9)	889 (84.0)	174 (86.6)	715 (83.4)
No answer	4 (0.4)	1 (0.9)	3 (0.4)	7 (0.7)	1 (0.5)	6 (0.7)
Employer provided gloves						
No	133 (14.6)	12 (10.2)	121 (15.3)	152 (14.4)	26 (12.9)	126 (14.7)
Yes	777 (85.3)	106 (89.8)	671 (84.6)	905 (85.5)	175 (87.1)	730 (85.2)
No answer	1 (0.1)	0 (0.0)	1 (0.1)	1 (0.1)	0 (0.0)	1 (0.1)
Employer provided eye shields						
No	444 (48.7)	52 (44.1)	392 (49.4)	518 (49.0)	94 (46.8)	424 (49.5)
Yes	466 (51.2)	66 (55.9)	400 (50.4)	539 (50.9)	107 (53.2)	432 (50.4)
No answer	1 (0.1)	0 (0.0)	1 (0.1)	1 (0.1)	0 (0.0)	1 (0.1)
Employer provided hand washing stations						
No	5 (0.6)	1 (0.8)	4 (0.5)	6 (0.6)	2 (1.0)	4 (0.5)
Yes	905 (99.3)	117 (99.2)	788 (99.4)	1051 (99.3)	199 (99.0)	852 (99.4)
No answer	1 (0.1)	0 (0.0)	1 (0.1)	1 (0.1)	0 (0.0)	1 (0.1)
Employer provided liquid soap and paper towels						
No	12 (1.3)	2 (1.7)	10 (1.3)	15 (1.4)	4 (2.0)	11 (1.3)
Yes	897 (98.5)	116 (98.3)	781 (98.5)	1041 (98.4)	196 (97.5)	845 (98.6)
No answer	2 (0.2)	0 (0.0)	2 (0.3)	2 (0.2)	1 (0.5)	1 (0.1)
Employer provided hand sanitizer						
No	70 (7.7)	8 (6.8)	62 (7.8)	89 (8.4)	20 (10.0)	69 (9.1)
Yes	840 (92.2)	110 (93.2)	730 (92.1)	968 (91.5)	181 (90.0)	787 (91.8)
No answer	1 (0.1)	0 (0.0)	1 (0.1)	1 (0.1)	0 (0.0)	1 (0.1)

© 2021 Mora AM et al. *JAMA Network Open.*

Workplace surfaces and tools regularly disinfected and kept clean						
No	94 (10.3)	12 (10.2)	82 (10.3)	115 (10.9)	17 (8.5)	98 (11.4)
Yes	785 (86.2)	98 (83.1)	687 (86.6)	905 (85.5)	175 (87.1)	730 (85.2)
No answer	32 (3.5)	8 (6.8)	24 (3.0)	38 (3.6)	9 (4.5)	29 (3.4)
Employer staggered breaks to reduce exposure						
No	494 (54.2)	66 (55.9)	428 (54.0)	583 (55.1)	112 (55.7)	471 (55.0)
Yes	413 (45.3)	51 (43.2)	362 (45.6)	469 (44.3)	89 (44.3)	380 (44.3)
No answer	4 (0.4)	1 (0.9)	3 (0.4)	6 (0.6)	0 (0.0)	6 (0.7)
Employer provided information on COVID-19 symptoms						
No	40 (4.4)	6 (5.1)	34 (4.3)	62 (5.9)	17 (8.5)	45 (5.3)
Yes	870 (95.5)	112 (94.9)	758 (95.6)	993 (93.9)	184 (91.5)	809 (94.4)
No answer	1 (0.1)	0 (0.0)	1 (0.1)	3 (0.3)	0 (0.0)	3 (0.4)
Employer provided information on how to protect themselves at work						
No	21 (2.3)	1 (0.8)	20 (2.5)	35 (3.3)	12 (6.0)	23 (2.7)
Yes	889 (97.6)	117 (99.2)	772 (97.4)	1020 (96.4)	189 (94.0)	831 (97.0)
No answer	1 (0.11)	0 (0.0)	1 (0.1)	3 (0.3)	0 (0.0)	3 (0.4)
Employer provided information on how to protect themselves at home and in the community						
No	47 (5.2)	3 (2.5)	44 (5.6)	67 (6.3)	17 (8.5)	50 (5.8)
Yes	863 (94.7)	115 (97.5)	748 (94.3)	988 (93.4)	184 (91.5)	804 (93.8)
No answer	1 (0.11)	0 (0.0)	1 (0.1)	3 (0.3)	0 (0.0)	3 (0.4)
Employer provided information on whom to call if they were sick						
No	99 (10.9)	18 (15.3)	81 (10.2)	129 (12.2)	27 (13.4)	102 (11.9)
Yes	811 (89.0)	100 (84.7)	711 (89.7)	926 (87.5)	174 (86.6)	752 (87.7)
No answer	1 (0.11)	0 (0.0)	1 (0.1)	3 (0.3)	0 (0.0)	3 (0.4)
Employer provided information on their ability to get free testing and treatment if they were sick						
No	237 (26.0)	31 (26.3)	206 (26.0)	289 (27.3)	62 (30.8)	227 (26.5)
Yes	672 (73.8)	87 (73.7)	585 (73.8)	765 (72.3)	138 (68.7)	627 (73.2)
No answer	2 (0.2)	0 (0.0)	2 (0.3)	4 (0.4)	1 (0.5)	3 (0.4)

© 2021 Mora AM et al. *JAMA Network Open.*

Employer provided information on where to get housing if they needed to quarantine or isolate away from home						
No	498 (54.7)	70 (59.3)	428 (54.0)	579 (54.7)	112 (55.7)	467 (54.5)
Yes	411 (45.1)	48 (40.7)	363 (45.8)	475 (44.9)	88 (43.8)	387 (45.2)
No answer	2 (0.22)	0 (0.0)	2 (0.3)	4 (0.4)	1 (0.5)	3 (0.4)
Employer provided information on the importance of staying away from work if they were sick						
No	55 (6.0)	5 (4.2)	50 (6.3)	77 (7.3)	15 (7.5)	62 (7.2)
Yes	854 (93.7)	113 (95.8)	741 (93.4)	976 (92.3)	185 (92.0)	791 (92.3)
No answer	2 (0.2)	0 (0.0)	2 (0.3)	5 (0.5)	1 (0.5)	4 (0.5)
Employer provided information on their benefit to get paid to stay away from work if they were sick						
No	263 (28.9)	36 (30.5)	227 (28.6)	316 (29.9)	58 (28.9)	258 (30.1)
Yes	646 (70.9)	82 (69.5)	564 (71.1)	738 (69.8)	142 (70.6)	596 (69.5)
No answer	2 (0.2)	0 (0.0)	2 (0.3)	4 (0.4)	1 (0.5)	3 (0.4)
Received education about COVID-19 from medical staff at workplace						
No	584 (64.1)	76 (64.4)	508 (64.1)	682 (64.5)	126 (62.7)	556 (64.9)
Yes	312 (34.2)	41 (34.8)	271 (34.2)	356 (33.6)	73 (36.3)	283 (33.0)
No answer	15 (1.7)	1 (0.8)	14 (1.8)	20 (1.9)	2 (1.0)	18 (2.1)

© 2021 Mora AM et al. *JAMA Network Open.*

eFigure. Correlation Heat Map of Risk Factors Associated With TMA and IgG Positivity Among Farmworkers, Monterey County, 2020 (n=1107)

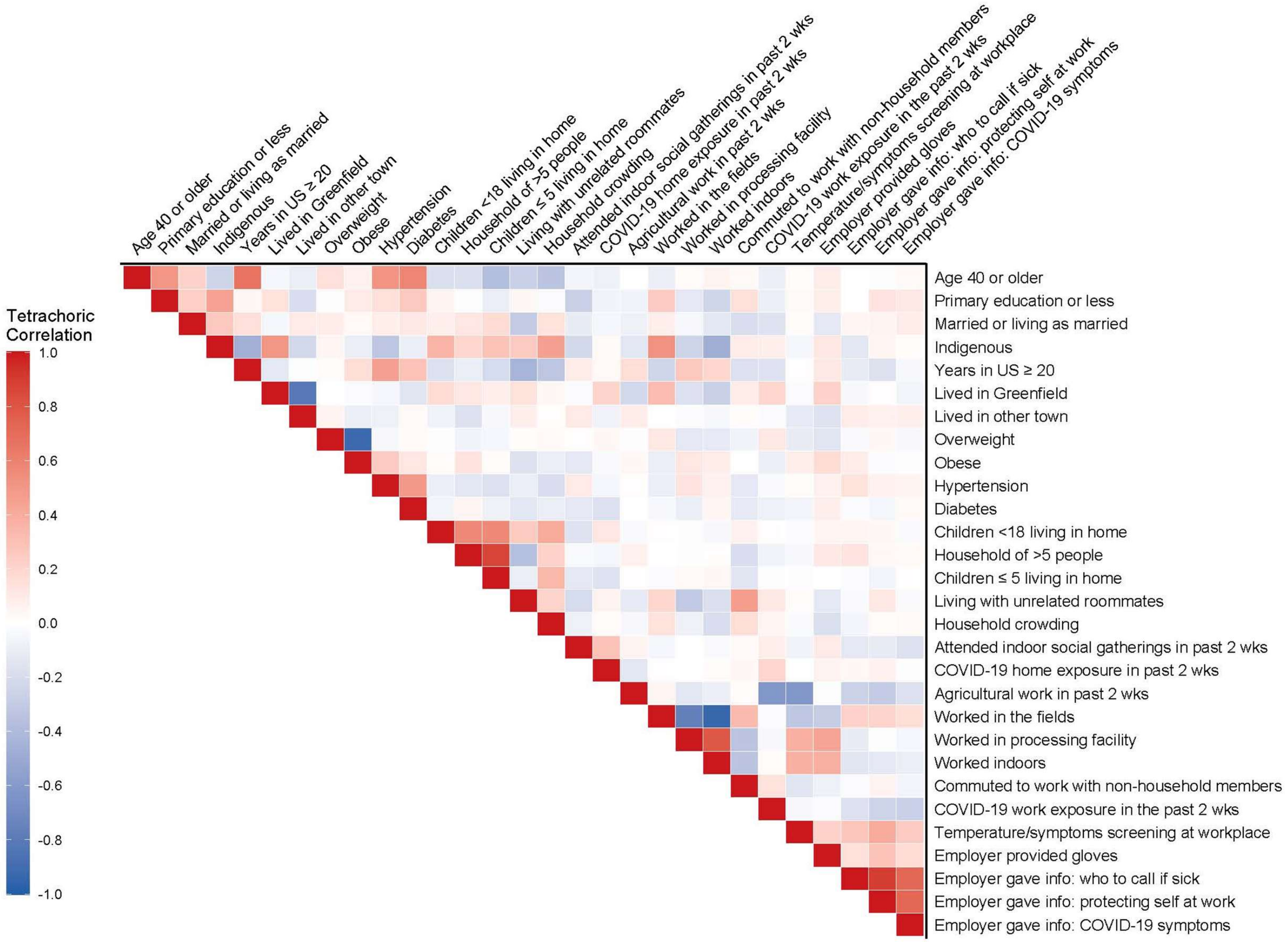

© 2021 Mora AM et al. *JAMA Network Open.*
