# Supplementary material for: Risk Factors Associated With SARS-CoV-2 Infection Among Farmworkers in Monterey County, California
Source: JAMA Netw Open. 2021 Sep 15;4(9):e2124116. doi: 10.1001/jamanetworkopen.2021.24116 (PMC8444020; doi:10.1001/jamanetworkopen.2021.24116)
Supplement: Supplement 2. — Nonauthor Collaborators [file jamanetwopen-e2124116-s002.pdf]

*Group Name(s): CHAMACOS-Project-19 Study Team							
***First Name and Middle Initial(s)**	***Last Name**	***Suffix (eg, Jr, III)**	Academic Degrees	Institution	Location (city, state/province, country)	Role or Contribution, eg, chair, principal investigator	Group (if more than 1 Group listed in the byline) and/or Subgroup (eg, Steering Committee)
Jose	Camacho		High school	Clinica de Salud del Valle de Salinas	Salinas, CA		
Gardenia	Casillas		BA	Clinica de Salud del Valle de Salinas	Salinas, CA		
Celeste	Castro		High school	Clinica de Salud del Valle de Salinas	Salinas, CA		
Madison J	de Vere		High school	University of California, Berkeley	Berkeley, CA		
Lupe	Flores		High school	Clinica de Salud del Valle de Salinas	Salinas, CA		
Lizari	Garcia		BA	Clinica de Salud del Valle de Salinas	Salinas, CA		
Maria	Reina Garcia		BS	Clinica de Salud del Valle de Salinas	Salinas, CA		
Terry	Gomez		High school	Clinica de Salud del Valle de Salinas	Salinas, CA		
Carly	Hyland		MS	University of California, Berkeley	Berkeley, CA		
Daniel	Lampert		BA	University of California, Berkeley	Berkeley, CA		
Aaron	McDowell-Sanchez		BS	Clinica de Salud del Valle de Salinas	Salinas, CA		
Dominic	Pina Montes		BA	University of California, Berkeley	Berkeley, CA		
Jacqueline	Montoya		High school	Clinica de Salud del Valle de Salinas	Salinas, CA		
Norma	Morga		High school	Clinica de Salud del Valle de Salinas	Salinas, CA		
Oguchi	Nkwocha		MS, MD	Clinica de Salud del Valle de Salinas	Salinas, CA		
Lilibeth	Nunez		High school	Clinica de Salud del Valle de Salinas	Salinas, CA		
Lizbeth	Ortiz-Pivaral		High school	University of California, Berkeley	Berkeley, CA		
Juanita "Liz"	Orozco		BS	Clinica de Salud del Valle de Salinas	Salinas, CA		
Marbel	Orozco		High school	California State University Monterey Bay	Seaside, CA		
Kimberly L	Parra		MPH	Clinica de Salud del Valle de Salinas	Salinas, CA		
Nargis	Rezai		High school	California State University Monterey Bay	Seaside, CA		
Maria T	Rodriguez		BS	Clinica de Salud del Valle de Salinas	Salinas, CA		
Monica	Romero		BA	Clinica de Salud del Valle de Salinas	Salinas, CA		
Hina	Sheth		RN	Clinica de Salud del Valle de Salinas	Salinas, CA		
Jon	Yoshiyama		MD	Clinica de Salud del Valle de Salinas	Salinas, CA		
Litzi	Zepeda		High school	Clinica de Salud del Valle de Salinas	Salinas, CA		
